# Evaluation of a Clinical Protocol–Guided Chatbot to Support Telecarers in Diabetes Management: Embedded Mixed Methods Study

**DOI:** 10.2196/91058

**Published:** 2026-10-23

**Authors:** Rebecca Hui Shan Ong, Hong Choon Oh, Bryan Peide Choo, Linette Zhi Ai Tan, Melissa Hui Wen Gunn, Jin Wee Ng, Anne Ching Ching Teng, Sharon Yu Bing Ong, Angela Hwee Koon Ng, Winnie Yi Ling Soo, Yian Chin Lee, Halimah Binte Sarim, Geok Choo Lee, Junaidah Binte Ibrahim, Kryslyn Mei Ying Kae, Jinyang Wu, Zhengyuan Liu, Mojisola Erdt, Siti Umairah Md Salleh, Siti Maryam Binte Ahmad Subaidi, Sakinah Binte Yusof, Nabilah Binte Md Johan, Kavineshaan Ramdas, Jia Shern Ang, Qi Yuan Tay, Yong Mong Bee, Nancy F Chen, Pavitra Krishnaswamy, Joan Joo Ching Khoo

**Affiliations:** 1 Health Services Research, Changi General Hospital, 2 Simei St 3529889, Singapore, 65 7888833; 2 Centre for Population Health Research and Implementation, SingHealth, Singapore; 3 Department of Endocrinology, Changi General Hospital, Singapore; 4 Regional Health System (Transition), Changi General Hospital, Singapore; 5 Department of Nursing Administrator, Changi General Hospital, Singapore; 6 Department of Care Coordination, Changi General Hospital, Singapore; 7 Telecare Unit, Changi General Hospital, Singapore; 8 Agency for Science, Technology and Research (A*STAR), Institute for Infocomm Research, Singapore; 9 Department of Endocrinology, Singapore General Hospital, Singapore

**Keywords:** type 2 diabetes mellitus, digital health, chatbot, tele-support, telecare, feasibility study

## Abstract

**Background:**

Type 2 diabetes mellitus (T2DM) is a chronic condition that requires ongoing self-management. While chatbots offer scalable monitoring and management, evidence on their integration into structured hospital tele-support workflows, where patient engagement and digital readiness may differ from those in standalone digital interventions, remains limited.

**Objective:**

This study aimed to describe the design of a clinical protocol–guided chatbot, Sharing Information with Users on Diabetes Actions and Interventions (*Siu Dai*), and evaluate its feasibility, usability, and acceptability among patients and telecarers within a hospital tele-support program.

**Methods:**

We used an embedded mixed methods design (QUAL [quan]) guided by the World Health Organization (WHO) monitoring and evaluating digital health interventions and the theoretical framework of acceptability. Through a co-design process involving clinicians, researchers, telecarers, and developers, the chatbot was aligned with established clinical protocols. It featured 7 precall sessions designed to collect patient data, generate summary reports for staff, and deliver targeted educational content. Patients (≥21-year-old individuals with T2DM) and telecarer nurses (with experience ≥6 mo) were recruited from an existing tele-support program. Data sources included interviews, online surveys (5-point Likert scale), and chatbot usage logs. Qualitative data were analyzed thematically; quantitative data were summarized descriptively to supplement and contextualize the qualitative findings.

**Results:**

Fourteen participants (7 patients and 7 telecarers) completed both interviews and surveys and were included in the analysis. Patients (mean age 45.3, SD 15.4 y) had a median diabetes duration of 10 (IQR 3-25; range 1‐30) years, and all were digitally literate. Telecarers had a median of 5 (IQR 4-9) years of telehealth experience. Quantitatively, patients showed high engagement, completing 79% of started sessions with a median completion time of 2 minutes and 58 seconds. Patient satisfaction was high, with 86% (6/7) willing to recommend the tool; however, telecarer endorsement for universal rollout to patients in other clinical programs or outpatient clinics was more cautious (3/7, 43%). Qualitative findings revealed 5 themes: (1) perceptions of chatbot usability, (2) alignment and integration into telecarers’ processes, (3) factors influencing acceptability of the chatbot, (4) perceived efficacy of the chatbot for T2DM self-management and care delivery, and (5) suggested improvements and enhancements. Integrated findings revealed that the chatbot was usable and functioned as a precall support tool that optimized the personalization of tele-support sessions. However, its perceived efficacy in self-management behavior was limited by this cohort’s extensive experience with T2DM management. Key barriers to adoption included administrative burden for telecarers due to a lack of electronic medical record integration and content redundancy for patients. Suggested enhancements included multilingual support and adaptive content catering to different levels of disease-specific literacy.

**Conclusions:**

A clinical protocol–guided chatbot is feasible for hospital tele-support, complementing rather than replacing human care. It automates routine assessments and reinforces education, but future iterations must prioritize workflow integration and adaptive content to sustain engagement.

## Introduction

Type 2 diabetes mellitus (T2DM) affects more than 500 million people worldwide, and its prevalence continues to rise [1]. In Singapore, the national prevalence of T2DM was 8.5% in 2022, with rates rising with age, from 1.9% in adults aged 30 to 39 years to more than 20% in those aged 60 to 74 years [2]. This places a burden on the health care system, particularly in regions with older populations such as eastern Singapore [3,4]. Poorly controlled T2DM can lead to complications, including cardiovascular disease, neuropathy, nephropathy, and retinopathy [5]. Effective management requires multifaceted interventions, with an emphasis on patient self-management, including lifestyle modification, glucose monitoring, and medication adherence [6-8].

Digital health tools, such as mobile apps and telehealth services, have shown promise in supporting self-management [7,9]. Among these, chatbots emerge as scalable tools to provide education, monitoring, and support between clinical encounters [10]. A recent meta-analysis reported modest but statistically significant glycated hemoglobin (HbA_1c_) reductions (~0.3%) with chatbot interventions [10], and users also valued the personalized feedback and emotional support features [11]. However, the overall evidence based on chatbot evaluation remains limited [10]. Furthermore, implementation challenges such as data accuracy, interoperability, and varying levels of digital literacy highlight the need for improved chatbot designs that are clinically credible and contextually appropriate [11-13].

Many existing diabetes chatbots rely on proprietary systems or general-purpose AI models, with limited reporting on adherence to evidence-based frameworks or clinical guidelines [10]. For instance, evaluations of ChatGPT as a tool for diabetes education showed omissions and inconsistencies in diabetes-related advice compared with established guidelines [14,15]. These findings highlight the risks of misinformation and the importance of aligning chatbots with a structured and validated care pathway.

Changi General Hospital (CGH), a tertiary hospital in Singapore, has a strong focus on T2DM management [16]. Its Health Management Unit (HMU), established in 2011, runs a tele-support program staffed by trained registered nurses known as telecarers. As part of the T2DM tele-support program, telecarers deliver 3 structured calls over 3 months, focusing on T2DM self-management, cardiovascular risk control, complication recognition, and lifestyle education, with content aligned to national and international guidelines. Preliminary evaluations of the HMU program have shown improvements in glycemic control [17].

However, sustaining such nurse-led telecare is increasingly difficult due to workforce constraints and growing patient demand amid population aging [18]. In response, we developed a clinical protocol–guided chatbot, Sharing Information with Users on Diabetes Actions and Interventions (*Siu Dai*), designed to augment HMU telecare. The name “Siu Dai” is a colloquial Singaporean term for “less sugar,” typically used when ordering beverages. This nomenclature was selected to provide a culturally familiar and approachable identity for the chatbot. It also served as a culturally resonant metaphor for the chatbot’s goal of supporting glycemic control and lifestyle modification. *Siu Dai* was not intended to replace human interaction but to support it. It had 3 core functions: (1) a precall assessment comprising 7 chatbot sessions to collect patient information before each telehealth call, enabling more focused telehealth conversations; (2) automatic generation of a summary report after each precall chat session for telecarers to review before the scheduled tele-support call and prioritize issues; and (3) delivery of educational coaching and tips within each chatbot session, triggered by the patient’s responses and selections. By automating routine data collection and reinforcing key messages, the chatbot aimed to help telecarers focus on more complex or relational aspects of care. This human-digital hybrid approach reflects the wider consensus that digital tools should augment rather than replace traditional care [19]. Despite the growing use of chatbots in diabetes care, evaluations that capture both quantitative metrics and lived user experiences within structured hospital workflows remain limited. Given this gap and the complexity of embedding a digital tool into a hospital-based tele-support program, relying on a single data source would be insufficient. While quantitative metrics can track chatbot engagement and clinical trends, they often fail to capture the nuanced experiences of patients and telecarers navigating these tools within existing care workflows. A mixed methods approach was therefore used, with the qualitative strand serving as the core component and quantitative data as the supplemental component to provide contextual breadth for evaluating the feasibility, usability, and acceptability of the chatbot among patients and telecarers.

## Methods

### Study Design

We conducted a single-arm feasibility and acceptability study using an embedded mixed methods design (QUAL [quan]). In this design, the qualitative strand served as the primary method of evaluation, while quantitative survey data and technical metrics provided supplementary context for the qualitative findings (Figure 1). The conduct and reporting of this study were guided by the Good Reporting of a Mixed Methods Study (GRAMMS) checklist [20]. The study was guided by 2 established frameworks: (1) the World Health Organization’s monitoring and evaluating digital health interventions (M&E DHI) framework for assessing usability and feasibility [21] and (2) the theoretical framework of acceptability (TFA) to evaluate constructs such as affective attitude, burden, coherence, perceived effectiveness, self-efficacy, and ethicality [22] (Table 1).

**Figure 1.**
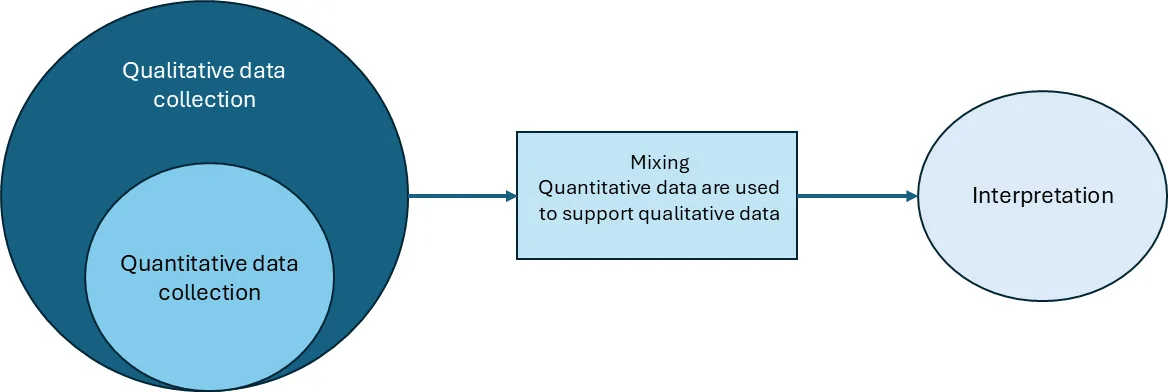
Embedded mixed methods design (QUAL[quan]).

**Table 1.** Overview of data collection methods, guiding frameworks, and focus areas.

Evaluation domain	Target group	Data source	Framework/instrument	
			TFA^a^	M&E DHI^b^
Usability	Patients	Online survey^c^, technical metrics, and semistructured interviews	Affective attitude, burden, ethicality, coherence, and self-efficacy	System reliability and usability
Usability	Telecarers	Online survey and semistructured interviews	Affective attitude, burden, and coherence	System reliability and usability
Integration and fit	Telecarers	Online survey and semistructured interviews	Affective attitude, burden, and coherence	Workflow integration
Acceptability	Patients	Online survey and semistructured interviews	Affective attitude, burden, ethicality, and coherence	Acceptability
Acceptability	Telecarers	Online survey and semistructured interviews	Affective attitude, burden, and coherence	Acceptability
Efficacy	Patients	Online survey and semistructured interviews	Affective attitude, burden, ethicality, perceived effectiveness, and self-efficacy	Early efficacy indicators
Efficacy	Telecarers	Online survey and semistructured interviews	Affective attitude, burden, and perceived effectiveness	Early efficacy indicators
Suggestions for improvements	Patients and telecarers	Semistructured interviews	Qualitative open-ended questions on suggestions for improvements	Qualitative open-ended questions on suggestions for improvements

a TFA: theoretical framework of acceptability.

b M&E DHI: monitoring and evaluating digital health interventions framework.

c Quantitative surveys used a 5-point Likert scale (1=strongly disagree, 5=strongly agree). Patient surveys included 22 items; telecarer surveys included 19 items.

### Setting and Participants

Participants were recruited via purposive sampling from the CGH HMU tele-support program. Patient inclusion criteria required individuals to be 21 years of age or older, have a clinical diagnosis of T2DM, be smartphone users, and demonstrate proficiency in English. Telecarers were eligible if they were registered nurses with a minimum of 6 months of experience in the HMU tele-support program and used the chatbot-generated summary reports during the study period.

### Study Procedures

The study was conducted from November 2023 to October 2024. Qualitative data were collected through semistructured interviews lasting approximately 45 to 60 minutes. Interviews were conducted in English, either in person or via Zoom, by researchers (RHSO and JWN) who were not involved in the participants’ direct clinical care or telecarers’ work to minimize social desirability bias. The interview guides were mapped to the TFA and M&E DHI domains. All sessions were audio-recorded and transcribed verbatim for analysis. Quantitative data were collected via online closed-ended surveys hosted on Qualtrics. The survey component is described according to the Checklist for Reporting Results of Internet E-Surveys (CHERRIES) [23]. A survey link was sent to enrolled participants, together with a unique participant code to identify unique responses. Participants were informed that the survey would take approximately 15 to 20 minutes to complete. The survey comprised 11 pages, with each page containing between 1 and 8 items. Survey completeness was ensured by using forced responses on all items. A back button was enabled to allow participants to review and change their answers. Survey items were self-developed by the research team and mapped to TFA and M&E DHI framework domains (Multimedia Appendix 1). Patients additionally self-reported their digital literacy status (assessed as very good/good/moderate/poor) and prior experience with using chatbot applications. Technical usage logs, including completion rates and interaction timestamps, were extracted from the chatbot database.

### Ethical Considerations

The SingHealth Centralized Institutional Review Board approved the study (reference 2022/2374). Written informed consent was obtained from all participants, and data were deidentified for analysis. All costs are reported in Singapore dollars (SGD). Participants who completed all study activities were given a token of appreciation (SGD 80; the exchange rate at the time of the study was approximately 1.00 SGD=US $0.74).

### Chatbot Co-Design and Development

The chatbot was co-designed with clinicians, telecarers, chatbot developers, system engineers, content designers, and researchers. Its conversation flow was derived from a retrospective review of 852 HMU telecare transcripts (17 telecarers and 173 patients), from which common questions, responses, and educational topics were extracted to build a clinically guided protocol aligned with existing HMU workflows and guidelines. The branching logic defining the follow-up questions, relevant advice, and educational content was co-designed with telecarers to ensure a coherent progression of questions based on each patient’s prior responses. Summary report templates were likewise co-designed with telecarers so that prioritized issues, follow-up flags, and key patient inputs could be easily reviewed without adding unnecessary preparation time before calls.

### System Architecture and Technical Build

The chatbot was developed as a message-based interactive agent system using Telegram Messenger (Android version 9.6.0; Apple iOS version 8.9), allowing patients to receive prompts, multimedia guidance, and reminders. The dialogue engine was built on the Botpress framework (version 12.30.9), which handled message parsing, semantic intent extraction, the recording of customized dialogue loops, and the maintenance of session continuity. All dialogue logic was predefined to ensure that responses adhered strictly to CGH tele-support protocols and clinical guidelines. An SQL-based dialogue and scheduling database within Botpress stored conversation data and patient metadata, while the dialogue scheduler coordinated communication between patients and telecarers, maintained timelines for the planned sessions, and triggered automated reminders. The system incorporated active engagement functions such as interactive answer-selection buttons with predefined response options and dynamic branching based on patient responses. The chatbot and its summary reports were hosted on Amazon Web Services (AWS) (Ubuntu version 22.04) with secure access (unique ID and password) and data handling within the AWS environment. All platforms used (Telegram, Botpress, and AWS) are commercially or publicly available, allowing other institutions to reproduce a similar architecture, subject to their own compliance and data governance requirements.

### Workflow and Content Delivery

Iterative prototyping and internal testing cycles with telecarers and an individual with T2DM in HMU led to refinements in usability, content accuracy, and report formatting prior to this pilot evaluation. These predeployment cycles were not a formal study, and the present study represents the chatbot’s first formal evaluation in a real-world tele-support context with patients. The chatbot workflow and question group distribution across the 7 sessions are detailed in Figure 2 and Multimedia Appendix 2, categorized by the tele-support schedule: (1) question group 1 preceded call 1, (2) question groups 2 to 4 preceded call 2, and (3) question groups 5 to 7 preceded call 3.

**Figure 2.**
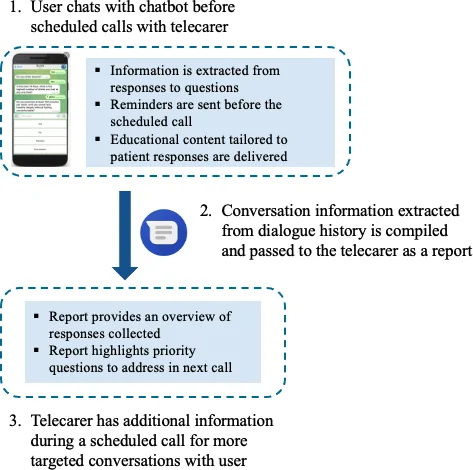
Chatbot workflow process.

### Sample Size Estimation

As a feasibility study, no formal sample size calculation was conducted. A target of 20 participants was set, aiming for at least 9 interviews across both patients and telecarers, consistent with empirical guidance on sample sizes for qualitative research [21,24,25] and with early-stage digital health feasibility pilots, where small qualitative samples are typical [11,26,27].

### Data Analysis

Qualitative transcripts were analyzed using thematic analysis in Dedoose [28]. Three coders (RHSO, LZAT, and MHWG) familiarized themselves with the 14 transcripts through repeated reading. To develop the codebook, 3 transcripts were coded by all 3 coders, prioritizing interpretive depth and analytical judgment over interrater reliability metrics. Discrepancies were discussed and reconciled through iterative team meetings, during which disputed codes were reviewed against the raw transcript excerpts and refined to best represent the data. This process informed the inductive formation of a preliminary codebook, which was also guided by deductive elements drawn from the TFA and M&E DHI constructs to maintain alignment with the study aims. The remaining transcripts were then divided among the 3 coders for independent coding, and themes and codes were further refined through iterative team discussions until final consensus was achieved. Mixed methods integration was conducted with the 14 participants (7 patients and 7 telecarers) who completed both phases, ensuring that integration drew on linked qualitative and quantitative data from the same individuals. The quantitative strand served a supplemental, descriptive role consistent with the QUAL (quan) design [29-31], while the qualitative transcripts were the primary focus of analysis [32]. We integrated the 2 datasets using a narrative approach and a joint display table [33], through which quantitative data supplemented and contextualized the qualitative themes across evaluation domains.

## Results

### Participant Characteristics

Of the 19 participants enrolled (11 patients and 8 telecarers), 5 were noncompleters who were uncontactable or unable to participate due to time constraints. Therefore, 14 participants (7 patients and 7 telecarers), who completed both the quantitative surveys and the interviews, formed the analytic sample for this study (Table 2). The patient cohort (n=7) had a mean age of 45.3 (SD 15.4) years and reported a median diabetes duration of 10 years, with an IQR of 3 to 25 years and a range of 1 to 30 years. All patients were identified as digitally literate and smartphone users, and 2 participants noted prior experience with chatbots. The telecarer group (n=7) consisted of female registered nurses with a median of 5 (IQR 4-9) years of experience in telehealth (range of 4‐12 y).

**Table 2.** Profile of 14 participants (7 patients and 7 telecarers) included in the analysis.

Characteristics	Patients (n=7)	Telecarers (n=7)
Age in years, mean (SD)	45.3 (15.4)	53.1 (14.5)
Years with T2DM^a^, median (SD; range)	10 (3-25; 1‐30)	—^b^
Years of telecare working experience, median (SD; range)	—	5 (4-9; 4-12)
Gender, n (%)		
Men	3 (43)	0 (0)
Women	4 (57)	7 (100)
Ethnicity, n (%)		
Chinese	4 (57)	5 (71)
Malay	1 (14)	2 (29)
Indian	2 (29)	0 (0)
Education, n (%)		
Primary or below	0 (0)	—
Secondary and pretertiary	3 (43)	—
Tertiary or above	4 (57)	—

a T2DM: type 2 diabetes mellitus.

b Not applicable.

### Chatbot Technical Metrics and Quantitative Feedback

The technical metrics for 7 patients showed a median session completion time of 2 minutes 58 seconds (IQR 2 min 12 s to 5 min 27 s). Patients completed 79% of the sessions, and 57% (n=4) completed all 7 sessions. Technical disruptions were minimal, with 3 time-outs recorded across the study period (1 in session 1, 1 in session 2, and 1 in session 5). Chatbot usage patterns indicated that 57% (n=4) of patients accessed at least one educational content item, and 86% (n=6) used the chatbot to flag specific topics for their upcoming tele-support calls.

All enrolled participants completed the quantitative survey, with no duplicate responses received. The surveys were also fully completed, with no incomplete items. Overall satisfaction was high among patients, with 86% (n=6) reporting they were satisfied and would recommend the chatbot. Telecarers’ findings were more mixed, with 43% (n=3) supporting a broader rollout of the system to patients in other HMU programs or outpatient clinics. The participants’ full survey responses are available in Multimedia Appendix 3.

### Integrated Findings

In the following sections, we present an integrated analysis of qualitative and quantitative findings across each evaluation domain (Table 3; Multimedia Appendix 4).

**Table 3.** Joint display of qualitative themes with supplementary quantitative findings.

Domain	Qualitative themes	Supplementary quantitative findings	Integration/interpretation
Usability	Theme 1: Perceptions of chatbot usability. Subtheme 1.1: Patients’ perceptions of chatbot usability. Subtheme 1.2: Telecarers’ perceived usability of the chatbot. Subtheme 1.3: Literacy and perceived burden of use.	Patients: 86% (6/7) could use the chatbot independently 86% (6/7) found it was easy to use 0% (0) felt there were too many questions. ·Telecarers: 86% (6/7) could review the chatbot report easily. 57% (4/7) found the data captured sufficient for planning calls.	The technical user interface of the chatbot was highly rated for usability, and the chatbot had a relatively short median usage time (2 min 54 s). But the qualitative data identified content fatigue and repetition as the primary drivers of user burden rather than technical complexity. Furthermore, while most telecarers (6/7, 86%) found the platform accessible, qualitative perceptions of data sufficiency reflected a gap between technical usability and functional utility.
Integration and fit	Theme 2: Alignment and integration of the chatbot into telecarers’ processes. Subtheme 2.1: Alignment with existing telecare workflows. Subtheme 2.2: Process and operational challenges.	Telecarers: 28% (2/7) agreed that the chatbot should be incorporated for all telecarers. 43% (3/7) agreed that the chatbot could be easily implemented in their workflow. 43% (3/7) preferred patients to use the chatbot prior to scheduled calls.	The qualitative data clarified that low rollout support is not a rejection of the chatbot technology itself, but a response to concerns about workflow integration. The low agreements seen in the quantitative data are explained by narrative accounts of the additional efforts required to integrate the chatbot data and scheduling into the existing telecarers’ processes.
Acceptability	Theme 3: Factors influencing patients’ acceptability of the chatbot. Subtheme 3.1: Concerns about data privacy and ethical use. Subtheme 3.2: Institutional trust and credibility. Subtheme 3.3: Preference for human touch and relational engagement. Subtheme 3.4: User readiness and capacity for chatbot engagement.	Patients: 0/7 had concerns about data privacy. 71% (5/7) trusted the chatbot advice. 57% (4/7) felt as comfortable communicating with the chatbot as with a telecarer. 57% (4/7) would continue using the chatbot. 86% (6/7) would recommend it to others.	Patients trusted and accepted the chatbot in part due to institutional branding. Concerns about data privacy were raised but were expressed by a minority. The lower comfort score in communicating with the chatbot as compared to a telecarer (4/7, 57%) is qualitatively explained by the preference for the human touch and the perceived inability of the chatbot to handle the relational and psychosocial aspects of T2DM^a^ care. A divergence was observed between personal intent to continue use (4/7, 57%) and the high rate of recommendation to others (6/7, 86%). Qualitative interviews suggested an explanation: many patients felt they were familiar with the content the chatbot had to offer because they were experienced and well-versed in managing their T2DM. However, they still viewed the chatbot as useful for others, specifically those who are newly diagnosed and still learning the basics.
Efficacy	Theme 4: Perceived efficacy of the chatbot on T2DM self-management and care delivery. Subtheme 4.1: Relevance and personalization of content. Subtheme 4.2: Support for self-management and behavior change. Subtheme 4.3: Enhancing care quality and service.	Patients: 86% (6/7) felt the educational content was relevant. 57% (4/7) reported improved understanding of T2DM. 43% (3/7) felt it improved their T2DM management. 86% (6/7) felt their chatbot inputs helped telecarers communicate effectively. Telecarers: 43% (3/7) reported improved quality of patient care. 57% (4/7) felt it helped patients better manage their condition. 43% (3/7) reported positive overall productivity impact. 71% (5/7) felt it enabled more personalized conversations. 43% (3/7) felt it aided patient prioritization.	The data suggested that the chatbot’s content was relevant, and its strength was in enabling more focused, personalized communication between patients and telecarers, rather than directly transforming self-management behaviors. Qualitative narratives suggested the chatbot optimized the precall processes, but as the patients in this study were already well-versed in managing their T2DM, the chatbot might not have shown as big of an impact as it would with newly diagnosed individuals. This might have limited the potential clinical and care efficacy of this study.

a T2DM: type 2 diabetes mellitus.

#### Usability

Analysis revealed high technical usability but highlighted content-related and linguistic barriers to broader adoption. Quantitatively, 86% (6/7) of patients found the chatbot interface easy to use, and 86% (6/7) of telecarers found the chatbot-generated report easy to review. Patients described the Telegram-based interface as familiar and convenient, and none reported that the number of chatbot questions was too many. However, qualitative findings identified a perceived burden, whereby some patients found the standardized question sets repetitive or lengthy over time:

*Some questions were repeated to see how honest we are. I know. I also do design survey questions…but some people will get frustrated, including me. You know, why they keep asking the same question.*

[P6, Patient]

*Sometimes I don't know when it’s [going to] end. It feels like it’s ongoing for very long.*

[P5, Patient]

Interviews also identified language proficiency as a potential barrier, with participants noting that an English-only chatbot might exclude non–English-speaking demographics.

Telecarers’ feedback highlighted a usability-utility gap. Qualitatively, telecarers found the chatbot dashboard intuitive to navigate, yet felt the standardized questions lacked the depth needed for tele-support care. This pattern was mirrored quantitatively; while 86% (6/7) of telecarers found the reports easy to review, only 57% (4/7) considered the data sufficient for call planning.

*Interviewer: Is there any additional information that the chatbot should collect that might help your work? Telecarer: Mmm...for whatever you all collect, I think at the end also we have to study the background of the patient…Before you call a patient, you must really read up about the background. What kind of job this person do? Does the person always eat out, eat at home? Who’s the one who do the cooking? I think the knowledge to the telecarer is very important...That's why our call to a patient must be personalized. It's not a standard call script, feed all my ten patients. No. This is a case by case.*

[T206, Telecarer]

#### Integration and Fit Into Telecarers’ Processes

Findings on workflow integration illustrated a nuanced relationship between technical ease and practical utility. At a functional level, the chatbot optimized tele-support interactions, with telecarers qualitatively reporting that having precollected data allowed for shorter, more focused calls. However, this benefit did not translate into a high level of integration, with only 29% (2/7) endorsing a universal rollout as part of the new workflow for all telecarers. The primary barrier was the lack of interoperability between the chatbot dashboard and the hospital’s primary electronic records. While the chatbot streamlined the conversation, the manual process of accessing separate reports created administrative work that neutralized the time saved during the call.

*I think the fact that I don't have to ask so much, the call gets shorter…But there’s no difference [to our workload] because there’s the additional (chatbot report) Excel that you have to open up, type, put back in. The workload is about the same.*

[T204, Telecarer]

Process and operational challenges emerged regarding the chatbot’s scheduling features, revealing a trade-off between outreach efficiency and workflow flexibility. On the one hand, telecarers noted that the scheduling feature reduced repetitive, unsuccessful call attempts by identifying suitable contact times.

*So at least I call at the correct time, not trying many times, to know which time is suitable.*

[T205, Telecarer]

However, telecarers also highlighted that these patient-preferred slots could clash with pre-existing clinical schedules, creating logistical difficulty.

*One thing I quite don’t like is the patient will request which day, what time [they want us to call]. Because sometimes we already promise other patients a certain time, so quite difficult.*

[T201, Telecarer]

Telecarers also observed that the patients using the chatbot were often proactive and knowledgeable about their T2DM management. While this demonstrated the chatbot’s potential in supporting a motivated patient base, the telecarers highlighted that it also presented future opportunities to tailor the chatbot’s outreach and design to better reach less engaged populations.

#### Acceptability

Acceptability was high, driven partially by institutional trust. Quantitatively, 71% (5/7) of patients trusted the advice due to its association with CGH.

*Cause I think this chatbot is developed by your side (CGH), so I think it has backing. All the information will have been verified all that, so I think it’s good.*

[P5, Patient]

Only a minority of patients expressed concerns related to data oversight and ethical use and, in some cases, reinforced a preference for human-mediated care.

*(I prefer) The telecarer. I think to a certain aspect, there’s always this concern about having information leaked when it’s online…I think all these kinds of platforms you can actually screenshot and then like share elsewhere.*

[P5, Patient]

Patients also highlighted their preference for the “human touch” and emphasized that the chatbot should supplement rather than replace telecarers. They highlighted the necessity of a human connection for navigating the emotional and complex aspects of diabetes support, a sentiment reflected in the quantitative data, where only 57% (4/7) of participants felt as comfortable communicating with the chatbot as with a telecarer.

*And the human link I guess, there is the tonality and the voice that it helps to soothe you so as well and also like “how you are doing today?” that kind of stuff, you know, to build rapport you need to ask questions to make the person feel comfortable. (The chatbot is just) like you know, right, okay, “what you need” that kind of stuff...Ya, the connectivity. I mean, it can imitate, but they won't be 100 percent…*

[P3, Patient]

The qualitative strand further highlighted that user readiness and capacity for engagement were contributing elements to a positive user experience and acceptability. Beyond the technical design of the chatbot, participants’ pre-existing literacy levels and their motivation to change and interact with a digital chatbot influenced their level of participation.

*I can tell [patient] really studied. He really got see the Siu Dai (chatbot). Then he talk to me, I can see he learn a lot also…He himself also keen to make changes. That’s the most important part. Patients (being) motivated.*

[T201, Telecarer]

#### Preliminary Perceived Efficacy

The chatbot’s efficacy was perceived by participants primarily as supporting T2DM self-management and facilitating tele-support call communication. Quantitatively, 86% (6/7) of patients felt the content was relevant and personalized, which was reflected in the qualitative data. Participants noted that the information was relevant and served as a reminder to help them stay mindful of their health goals between tele-support calls.

*So I find that the Siu Dai was very good cause it’s like it’s giving me like, oh, like, for example, it has like topics on how to maintain your diet, and then like how to recognize the hyper, like if you have symptoms as well, and also like then they will…They also remind me oh, the telecarer is going to call you, like you can select your date and time and everything like that. Then also it will ask whether are you on oral medication or insulin, so the question will be tailored to what type of like what type of medication you are taking, for example.*

[P3, Patient]

Qualitatively, telecarers and patients highlighted that the chatbot optimized tele-support by automating routine precall data collection. This allowed telecarers to bypass routine precall questions and transition to deeper clinical discussions.

*I can say is (the chatbot) cut down some of my questions, because I already have some basic understand about patient, so I no need to ask again…also make it more convenient.*

[T205, Telecarer]

*It’s like you have other question, (chatbot) AI is very limited, so you are unable to further in-depth question for clarity…So, I think the chatbot is like the initial interaction first, then when you have time to think about it, digest it, and the question that you want to ask, then the telecarer come in, so it goes hand in hand, so I find it’s very, very good.*

[P3, Patient]

This qualitative insight was corroborated by quantitative findings, where 71% (5/7) of telecarers agreed that the chatbot enabled more personalized conversations. While the chatbot was valued as a communication aid, only 43% (3/7) of patients and telecarers perceived improvements in T2DM management. Telecarers observed that many participants were already well-versed in T2DM self-management and engaged with their care. Patients and telecarers suggested that the chatbot’s efficacy might be more pronounced for newly diagnosed patients.

*The information provided there is very informative, but may not be so for me, because I read more than that, so it is like…It is good for someone who’s oblivious of what’s happening to their lives…Generally, it would be useful if you ask me, as a non-patient or a new patient.*

[P6, Patient]

Encouragingly, even within this cohort of well-versed patients with T2DM, the chatbot demonstrated potential to correct nutritional misconceptions. An illustrative case was observed with a patient who credited the chatbot’s educational content with a tangible shift in her dietary self-regulation:

*I think cause like usually I thought like fruits, healthy, can eat more. Then like after I see, they (the chatbot) say like, oh, certain fruits are high in sugar. I should take less…now I count (the number of grapes) then I eat.*

[P5, Patient]

#### Suggested Improvements and Enhancements to the Chatbot

Participants identified several areas for iterative development, including expanding the chatbot’s reach, improving accessibility, and enhancing personalization.

##### Social and Community Integration

Qualitative feedback highlighted the importance of community-based support. Participants suggested that the chatbot could involve caregivers. By granting caregivers access to educational content and monitoring data, the chatbot could facilitate a more holistic, household-centered approach to T2DM management.

##### Enhancing Accessibility and Engagement

To address the cognitive burden and linguistic barriers mentioned, participants recommended diversifying content delivery. Suggestions included replacing text-heavy modules with interactive infographics and videos to better serve users with varying literacy levels. Additionally, to improve engagement and flexibility, users proposed adding asynchronous messaging and conducting community roadshows to increase digital readiness and platform adoption.

##### Personalization and Scope Expansion

Participants also proposed content stratification. This involves tailoring modules to specific patient profiles (eg, newly diagnosed individuals vs individuals who are not newly diagnosed) and expanding the scope to include mental well-being and other comorbidities. This transition from a “one-size-fits-all” chatbot to a personalized health companion was viewed as essential for long-term sustainability.

## Discussion

### Principal Findings

This pilot evaluated the feasibility, usability, and acceptability of “*Siu Dai*,” a clinical protocol–guided chatbot designed to support T2DM self-management within a hospital tele-support program. While findings noted high patient acceptability and functional support for tele-support delivery, integration into clinical workflows showed mixed results.

Patients reported high technical usability, describing the interface as intuitive and convenient. However, the perception of the brief chatbot sessions as repetitive and lengthy may reflect the influence of content relevance and cognitive load on subjective time perception, whereby routine or low-novelty content leads to self-reported overestimation of duration rather than reflecting the objective length of the sessions [34,35]. Similarly, while telecarers found the automated chatbot summaries accessible, they identified a need for greater contextual granularity (eg, patients’ living and caregiving arrangements) to support clinical interpretation. These results indicate that while technical usability was present, future iterations must prioritize content and interaction design optimization to bridge the gap between usability and clinical utility. This tension reflects divergent priorities between patients, who favor brevity, and telecarers, who require depth for clinical decision-making. This conflict may not be fully resolvable through interface design alone, but instead requires deliberate trade-off decisions in future iterations.

The availability of precall responses enhanced the tele-support services by facilitating more focused dialogue and better prioritization of patients’ needs, but these gains were counterbalanced by increased administrative burdens, specifically telecarers’ concerns about having to navigate separate reports. This might have contributed to the limited endorsement for universal adoption among telecarers, echoing broader literature that highlights poor integration into existing health care systems as a key barrier to the adoption of digital health tools [36]. While initial co-design sessions with telecarers informed development, implementation uncertainty suggests that co-design must be a longitudinal, iterative process rather than a static predeployment phase, though achieving this in practice remains challenging due to resource, time, and technical constraints that are common in digital health implementation [37]. Future integration with electronic medical records (EMRs) might present an opportunity to resolve these interoperability hurdles and streamline data access, though such technical scaling was beyond the scope of this study.

Patient acceptability was high, partly anchored in the institutional credibility of CGH, a finding echoing the literature on systemic trust as a determinant for acceptance of health chatbots [38,39]. Despite this trust, participants highlighted the “human touch” as an irreplaceable component of care. Relational elements, such as empathy, emotional support, and the navigation of complex clinical nuances, were viewed by patients as beyond the current capabilities of the *Siu Dai* chatbot. This aligns with a growing consensus among users that digital tools are perceived as valuable adjuncts rather than clinical substitutes [36,40,41]. Our findings support this complementary care model and are consistent with the broader consensus in the digital health literature on the clinical use of chatbots, large language models, and AI-assisted care delivery [19,42], whereby routine tasks are automated to allow health care professionals to focus on complex clinical management and relationship-building. Importantly, this study provides empirical support for this principle within a hospital-based tele-support context.

Perceptions of efficacy were influenced by participants’ T2DM literacy levels and experiential knowledge. While newly diagnosed patients found the content helpful and instructive, those with extensive self-management experience noted content redundancy, a common critique among high-literacy users of digital health tools [43]. Telecarers expressed a similar professional ambivalence. They acknowledged the chatbot’s role in facilitating personalized dialogue but remained cautious regarding its impact on longitudinal clinical outcomes and care quality. Despite these reservations, both patients and telecarers recognized the chatbot’s efficacy in enhancing T2DM care-related dialogue and education. Additionally, the chatbot demonstrated “proof-of-concept” for behavioral nudging, as evidenced by a patient’s reported dietary modification following the chatbot’s advice, showcasing the promise of digital coaching in supporting behavior change in T2DM management [44]. Still, most participants viewed the chatbot as supplementary rather than transformative due to its one-size-fits-all approach. While clinical protocol–guided chatbots ensure standardized, evidence-based care delivery, they lack the algorithmic tailoring required to address personalized or complex care needs [36,45]. To move from a supplementary to a transformative role, future iterations must resolve this gap between standardized clinical protocols and users’ needs, ensuring that content remains relevant throughout the patient’s care journey.

### Implications for Practice and Research

#### Practical Implications

The results of this study provide implications for integrating digital tools like the Siu Dai chatbot into hospital-based tele-support. Institutional credibility remains important for adoption because trust in the chatbot’s content among patients was anchored in the hospital’s reputation. Future versions of the chatbot should leverage institutional branding to maintain acceptability and clearly communicate the chatbot’s clinical grounding and data security protocols to sustain patient trust and adoption. To ensure linguistic inclusivity within Singapore’s multiethnic population, multilingual support (English, Mandarin, Malay, and Tamil) will be crucial for broader engagement. Facilitated onboarding approaches, such as roadshows or in-clinic demonstrations, may help lower the initial barrier to entry for individuals with lower digital literacy or those hesitant to adopt the chatbot. The integration of audio and video content and, where technically feasible, voice-based interaction should be explored to further improve accessibility.

The study highlighted the need for content relevance that evolves with the user. For some patients, particularly those well-versed in T2DM management, the standardized content was perceived as repetitive, suggesting that adaptive tailoring may be needed to reduce redundancy and support sustained engagement. Proactively explaining the rationale for structurally repeated questions at session onset and streamlining questions based on patient and telecarer feedback may reduce perceived redundancy. A visible progress indicator (eg, percentage of chatbot questions completed) may also help by giving users a sense of how much is left and could make the session feel shorter. Framing the chatbot as a complement rather than a substitute for tele-support services aligns with patients’ preference for empathic human interaction. Telecarers can reinforce this by referencing chatbot outputs during calls, enabling a human-digital partnership that preserves the “human touch” required for emotional support and complex clinical discussions. Future iterations should also consider extending chatbot access to family caregivers, allowing them to access educational content and monitoring data alongside patients, thereby broadening the support network for T2DM self-management.

For sustainable clinical adoption, workflow efficiency is crucial. Clear protocols for reviewing chatbot-generated summary reports, streamlined data flow, and closer integration with EMRs, potentially via API-based solutions that surface chatbot data within existing clinical systems without requiring separate logins or manual data entry, may help reduce the administrative burden identified by telecarers. Beyond EMR integration, alternative approaches to surfacing patient-entered data within clinical workflows, such as generative AI-based summarization or survey tools interfaced with existing patient portal systems, may also warrant exploration in future iterations. Strengthening real-world buy-in will also require longitudinal co-design with telecarers beyond the initial design phase, ensuring that workflow integration evolves to support usability in routine practice.

#### Research Implications

Future research should explore hybrid chatbot architectures that combine structured, guideline-based content with adaptive AI components. In this study, the chatbot was deliberately designed to be clinically protocol-guided to ensure safety and adherence to guidelines. However, this approach limited conversational depth and adaptability. Participants expressed interest in features such as free-text input and more interactive content, reflecting broader trends toward the integration of large language models (LLMs) to support more personalized health dialogues [46-48]. Integrating LLMs with guideline-based frameworks may offer a balanced approach that preserves clinical fidelity while enhancing conversational quality and user engagement. A subsequent larger multisite pilot study should build on the lessons from this feasibility phase to refine chatbot content, test workflow integration solutions, and evaluate longitudinal engagement across a more diverse patient population.

### Strengths and Limitations

A primary strength of this study is the application of established digital health frameworks, the TFA and the M&E DHI, which contributed to the rigor of the evaluation. Additionally, the inclusion of both patients and telecarers provides a dyadic perspective that is often missing in early-stage chatbot research [10,36]. Third, the embedded mixed methods design (QUAL [quan]) allowed quantitative data to supplement and contextualize the qualitative findings. This integration addressed calls in the literature for more mixed methods research study designs to better understand the complexities of digital health in T2DM care [10]. However, limitations should also be noted. As a feasibility pilot study, clinical effectiveness was not assessed, and larger controlled trials are necessary to determine the impact on long-term T2DM outcomes. An additional limitation is that the patient sample was highly digitally literate, with all patients self-identifying as smartphone-proficient and digitally literate. This sampling characteristic may have positively skewed perceptions of usability and acceptability, and findings may not be generalizable to populations with lower digital literacy or limited smartphone access. Furthermore, technology comfort and technology anxiety were not assessed using validated instruments. Future studies should include such measures, as they may moderate perceptions of usability and acceptability. The survey items were developed for this study and have not undergone formal psychometric validation. Future work should assess their reliability and validity to strengthen measurement rigor. Lastly, the rapid evolution of AI and chatbot tools may outpace the current design.

### Conclusions

This pilot study suggests that a clinical protocol–guided chatbot can be feasibly embedded within a hospital tele-support program for T2DM management. Patients reported high usability and trust, and telecarers found chatbot-generated precall information helpful for structuring conversations. However, challenges related to workflow integration and limited content personalization remain key barriers to scaling. While chatbots cannot replace the empathy and relational aspects of human care, they may serve as valuable adjuncts by automating routine assessments and reinforcing education, enabling telecarers to focus on the more complex and relational dimensions of chronic disease management. Future refinements should prioritize smoother workflow integration, greater tailoring to individual needs, and a balanced use of automation to support a meaningful human-digital partnership in care.

## Supplementary material

10.2196/91058 Multimedia Appendix 1 Patient and telecarer survey.

10.2196/91058 Multimedia Appendix 2 Chatbot question group distribution across sessions.

10.2196/91058 Multimedia Appendix 3 Participants’ survey responses.

10.2196/91058 Multimedia Appendix 4 Themes, subthemes, and illustrative quotations.

## Abbreviations

AWS: Amazon Web Services
CGH: Changi General Hospital
CHERRIES: Checklist for Reporting Results of Internet E-Surveys
DHI: Monitoring and evaluating digital health interventions framework
EMR: electronic medical record
GRAMMS: Good Reporting of a Mixed Methods Study
HbA_1c_: glycated hemoglobin
HMU: Health Management Unit
LLM: large language model
T2DM: type 2 diabetes mellitus
TFA: theoretical framework of acceptability
WHO: World Health Organization
