# Supplementary material for: Evaluation of a Clinical Protocol–Guided Chatbot to Support Telecarers in Diabetes Management: Embedded Mixed Methods Study
Source: J Med Internet Res. 2026 Oct 23;28:e91058. doi: 10.2196/91058 (PMC13693827; doi:10.2196/91058)
Supplement: Supplementary file 1 [file jmir-v28-e91058-s001.docx]

# **Multimedia Appendix 1 –** Patient and telecarer survey

**Patient survey**

1. Gender
   a. Male
   b. Female
2. Age ________________years
3. Ethnicity
   a. Chinese
   b. Malay
   c. Indian
   d. Others: _______________
4. Education (please choose highest qualification achieved)
   a. No formal qualifications/lower primary
   b. Primary (PSLE)
   c. Secondary (‘O’/’N’ level)
   d. ITE/ NTC
   e. ‘A’ level/Polytechnic/diploma
   f. Bachelor’s degree
   g. Postgraduate Diploma
   h. Master’s degree
   i. Doctorate Degree
5. Perceived IT skills
   a. Very Good
   b. Good
   c. Moderate
   d. Poor
6. Have you used the internet as a source for health information?
   a. Yes
   b. No
   c. Unsure
7. Have you used a health chatbot prior to participation in this programme?
   a. Yes
   b. No
   c. Unsure
8. I am generally worried about my health
   a. Yes
   b. No
9. I am confident in identifying my own health problems
   a. Yes
   b. No
10. Is there anyone to help you in your diabetes care?
    a. Yes	(If yes, who: ________________)
    b. No
11. How long have you had diabetes?

________________years

12. How would you rate your understanding of diabetes?
    a. Good
    b. Fair
    c. Poor
13. Have you ever been diagnosed with the following conditions (More than one option can be selected)?
    a. High cholesterol
    b. High triglycerides
    c. High blood pressure
14. Have you asked another person to answer questions posed by “Siu Dai” on your behalf?
    a. Yes
    b. No
15. If you answered “Yes” for question 14, what were your reasons for letting another person answer the questions on your behalf?
    a. The question list was too lengthy
    b. Questions were difficult to understand
    c. The health chatbot platform was difficult to navigate
    d. Mobile phone screen or font size is too small for me
    e. I am not familiar with English
    f. Interruption by my personal or professional activities
    g. Interruption by the network issue and mobile phone in-coming calls
    h. Others (Indicate Reason __________________________________)
16. Have you ever left the conversation with “Siu Dai” midway through the session?
    a. Yes
    b. No
17. If you answered “Yes” for question 16, what were your reasons for dropping out?
    a. The question list was too lengthy
    b. Questions were difficult to understand
    c. The health chatbot platform was difficult to navigate
    d. Mobile phone screen or font size is too small for me
    e. I am not familiar with English
    f. The advice given by health chatbot is not giving appropriate
    g. Interruption by my personal or professional activities
    h. Others (Indicate Reason for dropping out __________________________________)
18. If you answered “Yes” for question 16, what was the frequency of you leaving the conversation with “Siu Dai” during this trial?
    a. 1 time
    b. 2 times
    c. 3 times
    d. 4 times
    e. > 4 times
19. The following statements are related to your perception of the reliability of “Siu Dai”. How strongly did you agree or disagree with the following statements?

|  | **Strongly agree** | **Agree** | **Neutral** | **Disagree** | **Strongly disagree** | **TFA construct** | **M&E DHI** |
| --- | --- | --- | --- | --- | --- | --- | --- |
| I feel “Siu Dai” is able to respond to my inputs with no delay |  |  |  |  |  | Burden | System reliability |
| I am able to enter my responses or inputs to “Siu Dai” with no delay |  |  |  |  |  | Burden | System reliability |
| “Siu Dai” is always online and accessible during the trial. |  |  |  |  |  | Burden | System reliability |

20. The following statements are related to your perception of the usability of “Siu Dai”. How strongly did you agree or disagree with the following statements?

|  | **Strongly agree** | **Agree** | **Neutral** | **Disagree** | **Strongly disagree** | **TFA construct** | **M&E DHI** |
| --- | --- | --- | --- | --- | --- | --- | --- |
| I am able to use “Siu Dai” independently without any assistance |  |  |  |  |  | Self-efficacy | Usability |
| I thought “Siu Dai” was easy to use |  |  |  |  |  | Burden | Usability |
| I think most people would find “Siu Dai” easy to use |  |  |  |  |  | Burden | Usability |
| I think that I would need the support of a technical person to be able to use “Siu Dai” effectively |  |  |  |  |  | Self-efficacy | Usability |
| I think “Siu Dai” platform had too many questions |  |  |  |  |  | Burden | Usability |

22. The following statements are related to your perception of the acceptability of the of “Siu Dai”. How strongly did you agree or disagree with the following statements?

|  | **Strongly agree** | **Agree** | **Neutral** | **Disagree** | **Strongly disagree** | **TFA construct** | **M&E DHI** |
| --- | --- | --- | --- | --- | --- | --- | --- |
| I find the questions in “Siu Dai” relevant to my current health concerns and problems |  |  |  |  |  | Intervention coherence | Efficacy |
| I find the educational content materials offered by “Siu Dai” to be relevant to me. |  |  |  |  |  | Intervention coherence | Efficacy |
| I am concerned about the privacy of my data when using “Siu Dai” |  |  |  |  |  | Ethicality | Acceptability |
| I trust the advice given to me by “Siu Dai” |  |  |  |  |  | Ethicality | Acceptability |
| I am comfortable with sharing my health conditions with “Siu Dai” |  |  |  |  |  | Ethicality | Acceptability |
| I would like to continue using “Siu Dai” to maintain or improve my health |  |  |  |  |  | Affective attitude | Acceptability |
| I am satisfied with “Siu Dai” |  |  |  |  |  | Affective attitude | Acceptability |

23. The following statements are related to effectiveness of “Siu Dai”. How strongly did you agree or disagree with the following statement?

|  | **Strongly agree** | **Agree** | **Neutral** | **Disagree** | **Strongly disagree** | **TFA construct** | **M&E DHI** |
| --- | --- | --- | --- | --- | --- | --- | --- |
| I find “Siu Dai” useful for me. |  |  |  |  |  | Perceived effectiveness | Efficacy |
| I feel “Siu Dai” has helped me to improve my understanding of my health condition |  |  |  |  |  | Perceived effectiveness | Efficacy |
| I feel “Siu Dai” has helped me better manage my diabetes condition |  |  |  |  |  | Perceived effectiveness | Efficacy |
| I feel as comfortable communicating with “Siu Dai” as speaking with a telecarer |  |  |  |  |  | Affective attitude | Acceptability |
| I am able to communicate my health problems better to the telecarer after using “Siu Dai” |  |  |  |  |  | Perceived effectiveness | Efficacy |
| I feel my inputs to “Siu Dai” have allowed the telecarers to communicate effectively with me over the scheduled calls |  |  |  |  |  | Perceived effectiveness | Efficacy |
| I would recommend “Siu Dai” to other patients in this diabetes telesupport programme |  |  |  |  |  | Affective attitude | Acceptability |

24. Do you have any suggestions on what features should be introduced to “Siu Dai” so that you will use it more often?

_____________________________________________________________________________

_____________________________________________________________________________

_____________________________________________________________________________

**Telecarer survey**

1. Gender
   a. Male
   b. Female
2. Age ________________years
3. Ethnicity
   a. Chinese
   b. Malay
   c. Indian
   d. Others: _______________
4. How many years have you been working as a telecarer?

_____________years

5. The following statements are related to reliability of “Siu Dai” for patients under your care. How strongly did you agree or disagree with the following statement?

|  | **Strongly agree** | **Agree** | **Neutral** | **Disagree** | **Strongly disagree** | **TFA construct** | **M&E DHI** |
| --- | --- | --- | --- | --- | --- | --- | --- |
| I am able to easily review the report generated by “Siu Dai” when I need to access it |  |  |  |  |  | Burden | Usability |
| I find the data collected by “Siu Dai” to be relatively complete (i.e. not much missing data) |  |  |  |  |  | Perceived effectiveness | Usability |

6. The following statements are related to the usability of “Siu Dai” for patients under your care. How strongly did you agree or disagree with the following statement?

|  | **Strongly agree** | **Agree** | **Neutral** | **Disagree** | **Strongly disagree** | **TFA construct** | **M&E DHI** |
| --- | --- | --- | --- | --- | --- | --- | --- |
| I prefer to have patients use “Siu Dai” prior to my scheduled calls |  |  |  |  |  | Intervention coherence | Integration & Fit |
| I think the patients find “Siu Dai” useful and helpful |  |  |  |  |  | Perceived effectiveness | Efficacy |
| I think the educational content provided in “Siu Dai” are useful for the patients. |  |  |  |  |  | Perceived effectiveness | Efficacy |
| I think the patients find “Siu Dai” platform easy to navigate |  |  |  |  |  | Burden | Usability |
| I find the data collected by “Siu Dai” is useful in planning and structuring my calls with patients |  |  |  |  |  | Perceived effectiveness | Usability |
| I feel it is easy to access and interpret the data collated from “Siu Dai” |  |  |  |  |  | Burden | Usability |

7. The following statements are related to your acceptability of “Siu Dai” for patients under your care. How strongly did you agree or disagree with the following statement?

|  | **Strongly agree** | **Agree** | **Neutral** | **Disagree** | **Strongly disagree** | **TFA construct** | **M&E DHI** |
| --- | --- | --- | --- | --- | --- | --- | --- |
| I feel “Siu Dai” should be incorporated as part of the new workflow for all telecarers |  |  |  |  |  | Intervention coherence | Integration & Fit |
| I feel that “Siu Dai” can be easily implemented within the workflow. |  |  |  |  |  | Burden | Integration & Fit |

8. The following statements are related to effectiveness of “Siu Dai” for patients under your care. How strongly did you agree or disagree with the following statement?

|  | **Strongly agree** | **Agree** | **Neutral** | **Disagree** | **Strongly disagree** | **TFA construct** | **M&E DHI** |
| --- | --- | --- | --- | --- | --- | --- | --- |
| I find “Siu Dai” has captured enough data for me to better plan my conversations with patients prior to scheduled calls |  |  |  |  |  | Perceived effectiveness | Efficacy |
| I feel that “Siu Dai” has positively impacted my communication with patients. |  |  |  |  |  | Perceived effectiveness | Efficacy |
| I find my overall productivity during the telesupport calls has improved by using “Siu Dai” |  |  |  |  |  | Perceived effectiveness | Efficacy |
| I find “Siu Dai” has helped patients better manage their diabetic condition |  |  |  |  |  | Perceived effectiveness | Efficacy |
| I feel “Siu Dai” improves the quality of patient care offered by the HMU programme |  |  |  |  |  | Perceived effectiveness | Efficacy |
| I am able to provide more personalised conversations with patients by using the report generated by “Siu Dai” |  |  |  |  |  | Perceived effectiveness | Efficacy |
| I am able to prioritise patients in greater need of attention for telephone follow-ups with the help of “Siu Dai” |  |  |  |  |  | Perceived effectiveness | Efficacy |
| I feel “Siu Dai” made a positive impact to my overall workflow |  |  |  |  |  | Perceived effectiveness | Integration & Fit |
| I would recommend “Siu Dai” be rolled out to patients in other HMU programme or clinical settings such as outpatient clinics |  |  |  |  |  | Affective attitude | Acceptability |

9. How should design in “Siu Dai” be revised so that it is useful to both patients and telecarers?

______________________________________________________________________________

______________________________________________________________________________

______________________________________________________________________________
