# Supplementary material for: Evaluation of a Clinical Protocol–Guided Chatbot to Support Telecarers in Diabetes Management: Embedded Mixed Methods Study
Source: J Med Internet Res. 2026 Oct 23;28:e91058. doi: 10.2196/91058 (PMC13693827; doi:10.2196/91058)
Supplement: Supplementary file 2 [file jmir-v28-e91058-s002.docx]

# Multimedia Appendix 2 – Chatbot question group distribution across sessions

|  | **Before tele-support call 1** | | **Before tele-support call 2** | | | **Before tele-support call 3** | | | |
| --- | --- | --- | --- | --- | --- | --- | --- | --- | --- |
| **Content** | **Chatbot session 1:**  **Question group 1** | **Chatbot session 2:**  **Question group 2** | | **Chatbot session 3:**  **Question group 3** | **Chatbot session 4:**  **Question group 4** | | **Chatbot session 5:**  **Question group 5** | **Chatbot session 6:**  **Question group 6** | **Chatbot session 7:**  **Question group 7** |
| Hba1c knowledge level | x^*^ |  | |  |  | |  |  |  |
| Vision symptoms |  | x | |  |  | |  |  |  |
| Hypoglycemic symptoms | x | x | |  |  | | x |  |  |
| Hyperglycemic symptoms | x | x | |  |  | | x |  |  |
| Diabetic medication - frequency and regiment | x | x | | x |  | |  | x |  |
| Blood glucose monitoring | x |  | | x |  | |  | x |  |
| Eye and feet check-up |  |  | |  | x | |  |  |  |
| Reference material (optional for patients to review) |  | x | | x |  | | x | x |  |
| Topics of interest for telecarer to discuss at next call | x |  | |  | x | |  |  | x |
| Blood pressure monitoring | x |  | |  | x | |  |  | x |
| Lifestyle-related questions - smoking, alcohol, lifestyle, diet |  |  | |  |  | |  |  | x |

*”x” denotes content covered during the respective chatbot sessions
