# Supplementary material for: Evaluation of a Clinical Protocol–Guided Chatbot to Support Telecarers in Diabetes Management: Embedded Mixed Methods Study
Source: J Med Internet Res. 2026 Oct 23;28:e91058. doi: 10.2196/91058 (PMC13693827; doi:10.2196/91058)
Supplement: Supplementary file 3 [file jmir-v28-e91058-s003.docx]

# Multimedia Appendix 3 – Participants’ survey responses

| Patients Survey Responses |  |  | |  | |  | |  | |
| --- | --- | --- | --- | --- | --- | --- | --- | --- | --- |
| Questions from Online Survey | Number of Responses for each Option on 5-point Likert Scale* | | | | | | | | |
|  | Option 1 (%) | | Option 2 (%) | | Option 3 (%) | | Option 4 (%) | | Option 5 (%) |
| I feel "Siu Dai" is able to respond to my inputs with no delay. | 2 (29) | | 3 (43) | | 1 (14) | | 1 (14) | | 0 (0) |
| I am able to enter my responses or inputs to "Siu Dai" with no delay. | 2 (29) | | 4 (57) | | 0 (0) | | 0 (0) | | 1 (14) |
| Siu Dai is always online and accessible during the trial. | 3 (43) | | 2 (29) | | 1 (14) | | 1 (14) | | 0 (0) |
| I am able to use "Siu Dai" independently without any assistance. | 5 (71) | | 1 (14) | | 0 (0) | | 0 (0) | | 1 (14) |
| I thought "Siu Dai" was easy to use. | 2 (29) | | 4 (57) | | 0 (0) | | 0 (0) | | 1 (14) |
| I think most people would find "Siu Dai" easy to use. | 2 (29) | | 4 (57) | | 0 (0) | | 0 (0) | | 1 (14) |
| I think that I would need the support of a technical person to be able to use "Siu Dai" effectively. | 0 (0) | | 0 (0) | | 2 (29) | | 2 (29) | | 3 (43) |
| I think "Siu Dai" platform had too many questions. | 0 (0) | | 0 (0) | | 5 (71) | | 1 (14) | | 1 (14) |
| I find the questions in "Siu Dai" relevant to my current health concerns and problems. | 2 (29) | | 2 (29) | | 3 (43) | | 0 (0) | | 0 (0) |
| I find the educational content materials offered by "Siu Dai" to be relevant to me. | 2 (29) | | 4 (57) | | 1 (14) | | 0 (0) | | 0 (0) |
| I am concerned about the privacy of my data when using "Siu Dai". | 0 (0) | | 0 (0) | | 3 (43) | | 3 (43) | | 1 (14) |
| I trust the advice given to me by "Siu Dai". | 2 (29) | | 3 (43) | | 2 (29) | | 0 (0) | | 0 (0) |
| I am comfortable with sharing my health conditions with "Siu Dai". | 2 (29) | | 5 (71) | | 0 (0) | | 0 (0) | | 0 (0) |
| I would like to continue using "Siu Dai" to maintain or improve my health. | 0 (0) | | 4 (57) | | 3 (43) | | 0 (0) | | 0 (0) |
| I am satisfied with "Siu Dai". | 1 (14) | | 5 (71) | | 1 (14) | | 0 (0) | | 0 (0) |
| I find "Siu Dai" useful for me. | 1 (14) | | 3 (43) | | 3 (43) | | 0 (0) | | 0 (0) |
| I feel "Siu Dai" has helped me to improve my understanding of my health condition. | 0 (0) | | 4 (57) | | 3 (43) | | 0 (0) | | 0 (0) |
| I feel "Siu Dai" has helped me better manage my diabetes condition. | 0 (0) | | 3 (43) | | 4 (57) | | 0 (0) | | 0 (0) |
| I feel as comfortable communicating with "Siu Dai" as speaking with a telecarer. | 0 (0) | | 4 (57) | | 1 (14) | | 2 (29) | | 0 (0) |
| I am able to communicate my health problems better to the telecarer after using "Siu Dai". | 1 (14) | | 3 (43) | | 3 (43) | | 0 (0) | | 0 (0) |
| I feel my inputs to "Siu Dai" have allowed the telecarers to communicate effectively with me over the scheduled calls. | 1 (14) | | 5 (71) | | 1 (14) | | 0 (0) | | 0 (0) |
| I would recommend "Siu Dai" to other patients in this diabetes telesupport programme. | 1 (14) | | 5 (71) | | 1 (14) | | 0 (0) | | 0 (0) |

*Scale options: 1 = Strongly Agree, 2 = Agree, 3 = Neutral, 4 = Disagree, 5 = Strongly Disagree

| Telecarers Survey Responses |  | | | | |
| --- | --- | --- | --- | --- | --- |
| Questions from Online Survey | Number of Responses for each Option on 5-point Likert Scale* | | | | |
| I am able to easily review the report generated by “Siu Dai” when I need to access it. | 0 (0) | 6 (86) | 1 (14) | 0 (0) | 0 (0) |
| I find the data collected by “Siu Dai” to be relatively complete (i.e. not much missing data). | 0 (0) | 4 (57) | 3 (43) | 0 (0) | 0 (0) |
| I prefer to have patients use “Siu Dai” prior to my scheduled calls. | 0 (0) | 3 (43) | 3 (43) | 1 (14) | 0 (0) |
| I think the patients find “Siu Dai” useful and helpful. | 0 (0) | 3 (43) | 4 (57) | 0 (0) | 0 (0) |
| I think the educational content provided in “Siu Dai” are useful for the patients. | 0 (0) | 6 (86) | 1 (14) | 0 (0) | 0 (0) |
| I think the patients find “Siu Dai” platform easy to navigate. | 0 (0) | 4 (57) | 3 (43) | 0 (0) | 0 (0) |
| I find the data collected by “Siu Dai” is useful in planning and structuring my calls with patients. | 0 (0) | 4 (57) | 3 (43) | 0 (0) | 0 (0) |
| I feel it is easy to access and interpret the data collated from “Siu Dai”. | 0 (0) | 3 (43) | 3 (43) | 1 (14) | 0 (0) |
| I feel “Siu Dai” should be incorporated as part of the new workflow for all telecarers. | 0 (0) | 2 (29) | 4 (57) | 1 (14) | 0 (0) |
| I feel that “Siu Dai” can be easily implemented within the workflow. | 0 (0) | 3 (43) | 3 (43) | 1 (14) | 0 (0) |
| I find “Siu Dai” has captured enough data for me to better plan my conversations with patients prior to scheduled calls. | 0 (0) | 4 (57) | 3 (43) | 0 (0) | 0 (0) |
| I feel that “Siu Dai” has positively impacted my communication with patients. | 0 (0) | 3 (43) | 4 (57) | 0 (0) | 0 (0) |
| I find my overall productivity during the telesupport calls has improved by using “Siu Dai”. | 0 (0) | 3 (43) | 4 (57) | 0 (0) | 0 (0) |
| I find “Siu Dai” has helped patients better manage their diabetic condition. | 0 (0) | 4 (57) | 3 (43) | 0 (0) | 0 (0) |
| I feel “Siu Dai” improves the quality of patient care offered by the HMU programme. | 0 (0) | 3 (43) | 4 (57) | 0 (0) | 0 (0) |
| I am able to provide more personalised conversations with patients by using the report generated by “Siu Dai”. | 0 (0) | 5 (71) | 2 (29) | 0 (0) | 0 (0) |
| I am able to prioritise patients in greater need of attention for telephone follow-ups with the help of “Siu Dai”. | 0 (0) | 3 (43) | 4 (57) | 0 (0) | 0 (0) |
| I feel “Siu Dai” made a positive impact to my overall workflow. | 0 (0) | 3 (43) | 2 (29) | 2 (29) | 0 (0) |
| I would recommend “Siu Dai” be rolled out to patients in other HMU programme or clinical settings such as outpatient clinics. | 0 (0) | 3 (43) | 2 (29) | 2 (29) | 0 (0) |

*Scale options: 1 = Strongly Agree, 2 = Agree, 3 = Neutral, 4 = Disagree, 5 = Strongly Disagree
