# Supplementary material for: Evaluation of a Clinical Protocol–Guided Chatbot to Support Telecarers in Diabetes Management: Embedded Mixed Methods Study
Source: J Med Internet Res. 2026 Oct 23;28:e91058. doi: 10.2196/91058 (PMC13693827; doi:10.2196/91058)
Supplement: Supplementary file 4 [file jmir-v28-e91058-s004.docx]

# Multimedia appendix 4 - Themes, subthemes and illustrative quotations.

| Themes | Subthemes | Illustrative quotations |
| --- | --- | --- |
| Perceptions of chatbot usability | 1.1: Patients’ perceptions of chatbot usability | *“I had a pause because I was engaged in something… when I came back, there was a time out. So that time out, once it happened, I had to restart back to question one… so that may deter people from going through the whole thing again.” — P4, Patient* |
|  | 1.2: Telecarers’ perceived usability of the chatbot | *“Before you call a patient, you must really read up about the background. What kind of job this person do? Does the person always eat out, eat at home? Who’s the one who do the cooking? I think the knowledge to the telecarer is very important.” — T206, Telecarer* |
|  | 1.3: Literacy and perceived burden of use | *"Sometimes I don't know when it's [going to] end. It feels like it's ongoing for very long." - P5, Patient* |
| Alignment and integration into telecarers’ processes | 2.1: Alignment with existing telecare workflows | *“With the chatbot is easier… you all already go through a layer that you pump in the question to them, then they answer them, we just refer [to the answers].” —* T206, Telecarer |
|  | 2.2: Process and operational challenges | *“One thing I quite don't like is the patient will request which day, what time [they want us to call]. Because sometimes we already promise other patients a certain time, so quite difficult.” —* T201, Telecarer |
| Factors influencing acceptability of the chatbot | 3.1: Concerns about data privacy and ethical use | *“(I prefer) The telecarer. I think to a certain aspect, there's always this concern about having information leaked when it's online… I think all these kinds of platforms you can actually screenshot and then like share elsewhere.” — P5, Patient* |
|  | 3.2: Institutional trust and credibility | *“Cause I think this chatbot is developed by your side (CGH), so I think it has backing. All the information will have been verified all that, so I think it's good.” — P5, Patient* |
|  | 3.3: Preference for human touch and relational engagement | *“..And the human link I guess, there is the tonality and the voice that it helps to soothe you so as well and also like "how you are doing today?" that kind of stuff, you know, to build rapport you need to ask questions to make the person feel comfortable. (The chatbot is just) like you know, right, okay, "what you need" that kind of stuff..Ya, the connectivity. I mean, it can imitate, but they won't be 100 percent...” — P3, Patient* |
|  | 3.4: User readiness and capacity for chatbot engagement | *“The chatbot is a technology only. It can help us only, but the initial thought should come from me... I should, I should have the mindset that it is tracking me and it's for my own health. If that thought is there in me, then this will be a 100 percent useful to me because it's helping me to be good like, know, live a healthy life. So it should start from me.” — P10, Patient* |
| Perceived efficacy of the chatbot on DM self-management and care delivery | 4.1: Relevance and personalization of content | *“Siu Dai (chatbot) all the questions is […] all the knowledge is inside. All the info, all the questions, like what we talk to patient all inside.” – T201, Telecarer*  *“I think the chatbot didn't help me.. maybe it help other people. I think it doesn't add any value to me using it… I would have expected that in the follow up call right, I should be able to tell them something of value to me. No, that's not within the so-called parameters of this Siu Dai chatbot and the telecare, which I thought was a missed opportunity.” — P1, Patient* |
|  | 4.2: Support for self-management and behavior change | *“I think cause like usually I thought like fruits, healthy, can eat more. Then like after I see, they (the chatbot) say like, oh, certain fruits are high in sugar. I should take less… now I count (the number of grapes) then I eat.” — P5, Patient*  *“The chatbot doesn't help me make (health) decision. It informs me of what resources are out there, but it's not relevant in the moment. You ask me the question, so do you have high glucose in the last one or two weeks or something? And I say yes, and it says, oh, then you should go and read this. I was thinking okay, well, that (high glucose) was two weeks ago.” — P1, Patient* |
|  | 4.3: Enhancing care quality and service | *“I can say is (the chatbot) cut down some of my questions, because I already have some basic understand about patient, so I no need to ask again… also make it more convenient.” — T205, Telecarer*  *“It's like you have other question or whatever, (chatbot) AI is very limited, so you are unable to further in-depth answer for clarity… So when the telecarer call you, you already have a question in mind. Oh, I want to ask this question... That kind of thing, so you can be more in depth. So, I think the chatbot is like the initial interaction first, then when you have time to think about it, digest it, and the question that you want to ask, then the telecarer come in, so it goes hand in hand, so I find it's very, very good.” — P3, Patient* |
| Suggested Improvements and Enhancements to the Chatbot | 5.1: Social and Community Integration | *"You need to also involve this group of caregivers […] Share with them [the information from the chatbot], because while they are giving care, they may not know all the implications and consequences of not having managed care."  — P1, patient* |
|  | 5.2: Enhancing Accessibility and Engagement | *"Show me charts, show me videos […] Tik Tok is so popular, and Reels, and Instagram, and all that stuff. Use that medium. Because I think in some cases some of us are not that literate... I truly believe in the power of visuals." — P1, patient*  *"It's good if Siu Dai (chatbot) can enable us to, for those who have no time to answer our call, just take our message.” — T203, Telecarer*  *"Patients need to download Telegram, then download Siu Dai (chatbot)... Their phone, is it up to date? You need to educate. Education goes a long way. If you can have a roadshow or something, when in the hospital to help them download it. You have to really go out there and help them."* — *P3, patient* |
|  | 5.3: Personalization and Scope Expansion | *"If you roll out that chatbot there's another thing that you need to include, which is the stratification of the participant. So, the beginner is given one set of videos. The middle guy is given this, and so on and so forth. So, you need to narrow it down to your target audience. It cannot be a one-for-all, you need to be able to narrow it down to them." - P1, patient*  *"I think it will add another dimension if it's adding in the psychiatric care. Like, 'hey, I'm doing badly with work right now, and it's causing me to increase food intake and all that. I really don't know how I can handle.” — P11, patient* |
